# Emergency Undocking Curriculum in Robotic Surgery

**DOI:** 10.7759/cureus.4321

**Published:** 2019-03-26

**Authors:** Derek A Ballas, Megan Cesta, David Gothard, Rami Ahmed

**Affiliations:** 1 Obstetrics and Gynecology, Summa Health System, Akron, USA; 2 Minimally Invasive Gynecologic Surgery, University of Louisville, Louisville, USA; 3 Clinical Research, Summa Health System, Akron, USA; 4 Emergency Medicine, Indiana University School of Medicine, Indianapolis, USA

**Keywords:** emergency undocking, robotic surgery, simulation, curriculum

## Abstract

Introduction

Traditional instruction for robotic surgery is typically devoid of training that addresses the delineation of interprofessional roles for operating room personnel. An emergency undocking scenario was developed for robotic surgeons with the objectives of improving time to access the patient, provider knowledge of and confidence in emergency undocking, completion of predetermined critical actions, and delineation of operating room personnel roles.

Methods

Over one month, participants joined in three sessions: Session 1 - formative, Session 2 - review, and Session 3 - summative. Embedded standardized participants (ESPs) represented members of the interprofessional team. Prior to entering the operating room for Sessions 1 and 3, trainees were asked to complete a confidence survey and multiple choice questionnaire (MCQ) for knowledge assessment. Participants were randomized to one of two cases and participated in the reciprocal case for the final session four weeks later. Following Session 1, participants underwent an educational intervention, including the proper technique for emergency undocking, emphasis on operating room personnel roles, and hands-on practice. Obstetrics and Gynecology (OBGYN) residents in post-graduate Years 2-4 and attending physicians with robotics privileges at Summa Health Akron Campus or Cleveland Clinic Akron General Medical Center were invited to participate. A total of 21 participants enrolled and finished the study.

Results

Among the 21 participants, there was a significant increase in the baseline level of knowledge (p-value=0.001) and in the confidence of surgeons when faced with an emergency undocking after the completion of our curriculum (p-value=0.003). Additionally, an improvement in the undocking times (p-value<0.001) and an increase in the critical actions performed (p-value=0.002) were observed.

Conclusion

The results of this study demonstrate that incorporating this curriculum into the training programs of robotic surgeons is an effective way to improve the surgical skill of emergency undocking.

## Introduction

Robot-assisted surgery is a rapidly changing field in gynecologic surgery [1]. Skill acquisition for many minimally invasive gynecologic surgeons and resident trainees is often viewed as inadequate due to the lack of a standardized training curriculum [2]. Instruction typically consists of completed online course work and a time period of proctored cases by a credentialed robotic surgeon. Emergency undocking is an infrequent event that may not occur at any time during a physician’s career, and the robotic surgeon may receive little to no training in the steps necessary to complete this procedure. With the increasing use of robot-assisted surgery, the frequency of critical incidents during surgery may increase [3]. In a situation in which emergency undocking is performed, the coordination of the entire interprofessional team involved in the operating room is required. There is a paucity of literature available outlining protocols or training for this procedure [4]. A team generally consists of a circulating nurse, scrub nurse, anesthesiologist, bedside assist surgeon, and robot console surgeon.

Despite an abundance of personnel and equipment in the operating suite, there is a lack of experience in managing these emergency situations [3,5]. To improve robotic crisis management, simulator-training scenarios have been developed [3,6-7]. Simulation has been used to validate checklists to improve surgical care [8]. Surgical training, in general, is becoming increasingly complex and time-restricted. In efforts to partially compensate for this and an often otherwise reduced volume of surgical training material, increasing emphasis is being placed on simulation [9]. It is well-established that simulation is the way forward as an effective alternative method of surgical education that helps bridge the gap created by these concerns [10].

In the event that an emergency undocking procedure is needed during robotic surgery, specific steps need to be employed in order to safely perform this task. Limited access to the patient can delay the start of effective measures to treat a life-threatening emergency and, in some cases, has resulted in fatality [3,11]. It is well-established that clear, concise communication is paramount during surgical emergencies for the benefit of patients and operating room staff [4,12-13]. The need for a protocol has been well-described in the robotics field since many practicing robotic surgeons have not encountered this situation in their training. However, there is limited literature outlining such protocols [4]. By standardizing the procedure for all robotic surgeons, best-care practices can be established.

Our aim is to have robotic surgeons undergo a specific training curriculum to assess their performance, knowledge, and confidence during an emergency situation and determine the effectiveness of the surgical training curriculum. The curriculum will focus on the surgeon’s ability to delineate well-defined and established roles in an emergency scenario in order to minimize the time from onset of injury to intervention and maximize the effective use of personnel. Our goal with this curriculum is to improve the confidence, knowledge, and proficiency of the steps necessary for a robotic surgeon to lead an interprofessional team.

## Materials and methods

Study location and equipment

**Figure 1:**
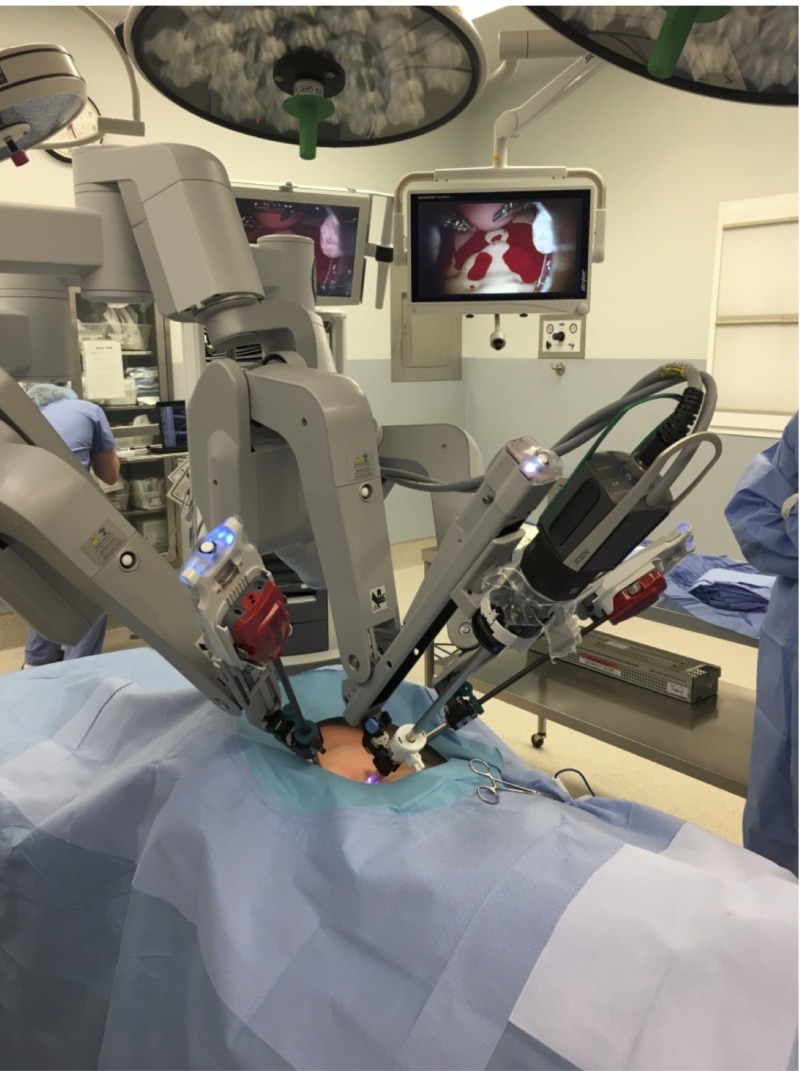
Robotic bedside cart and torso draped in steep Trendelenburg

The study took place at Summa Health, Akron Campus. The study was submitted to the Institutional Review Board and qualified for exempt status. Simulation assessment and training occurred in one of two operating suites dedicated to robotic surgery at the institution in an in-situ environment. The da Vinci Si system (Intuitive Surgical, California, US) was used for the simulation. Da Vinci training arms, as well as a 12 mm three-dimensional laparoscope, was used for the case. The modified ZOE Gynecologic Simulator by Gaumard® (Gaumard Scientific, Florida, US) was draped in a steep Trendelenburg position, as would be the case for most gynecologic laparoscopic surgeries (Figure 1).

**Figure 2:**
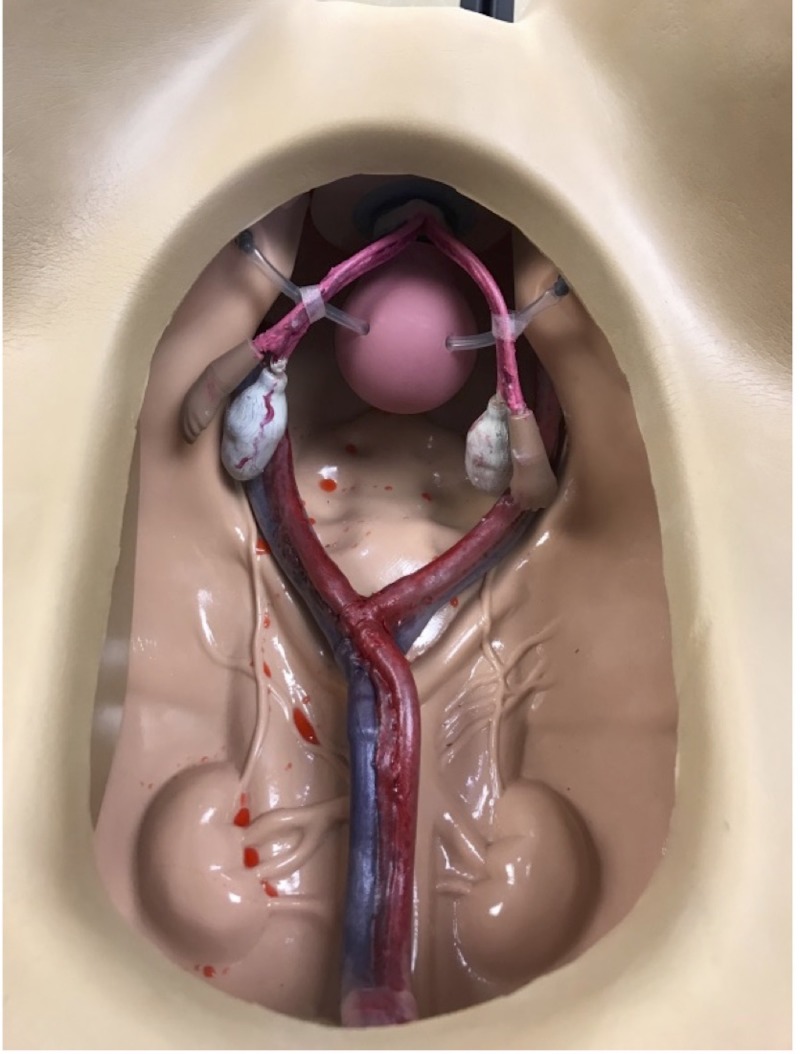
Modified training torso

The training torso was modified using a chest tube hooked to pressure bags filled with red food-colored intravenous (IV) fluid (Figure 2).

At the distal end of the tubing, a notch was created to allow for simulated bleeding in the case of a vessel injury. Manual pumping of the IV bag allowed for pulsatile release.

Curriculum development and outline

Participants attended three sessions, which included one formative simulation, one review session, and one summative simulation. The simulations involved the emergency undocking of the da Vinci Si robot (Intuitive Surgical, California, US) in an operating room setting, with a multidisciplinary team consisting of embedded standardized persons (ESPs) to fulfill the roles of a circulating nurse, scrub nurse, anesthesiologist, and bedside-assist surgeon. The two simulations were separated by approximately four weeks in order to ascertain the retention of knowledge. A skills session on proper emergency undocking was given during a debriefing following the first session. A survey was created using the Likert scale to determine confidence, and two credentialed robotic surgeons developed the multiple choice questionnaire (MCQ) questions based on available literature. To objectively assess individual pre- and post-curricular knowledge, all participants were asked to answer a 15-question MCQ before the initial formative simulation and after the summative simulation. To evaluate the impact of this curriculum on confidence, participants were asked to use a 15-question Likert scale, ranging from 1 to 5 (1 = very uncomfortable, 2 = somewhat uncomfortable, 3.= neutral, 4 = somewhat comfortable, and 5 = very comfortable) to rate their pre- and post-curriculum confidence for performing certain actions for emergency undocking. During simulations in Sessions 1 and 3, critical actions of emergency undocking of the da Vinci Si system (as established by da Vinci Si Surgical Systems) were recorded for grading by a fellowship-trained robotic surgeon. The time from the recognition of the need for emergency undocking to laparotomy was recorded. Data analysis included a nonparametric analysis of confidence scores and statistical student’s t-test analysis of knowledge assessment scores and critical action scores and trends in time were compared.

Participants, faculty, and staff

Obstetrics and Gynecology (OBGYN) residents and attending physicians with robotics privileges at Summa Health, Akron Campus, or Cleveland Clinic, Akron General Medical Center, were invited to participate in the training. All participants (residents and attending physicians) must have successfully completed the da Vinci online training modules for the da Vinci Si system. The skill levels of the participants ranged from novice to expert. The attending physicians' training levels included general gynecologist, urogynecologist, and gynecology oncologist. Resident physicians in post-graduate years two through four who completed the previously mentioned da Vinci training modules met inclusion criteria and were included. Participants were randomized in the order of case completion using the Microsoft Office Excel RAND function (Microsoft Corporation, Washington, US). Faculty included a fellowship-trained robotic surgeon, a medical simulation expert, and a medical simulation fellow with a background in gynecology and robotic surgery. ESPs fulfilled operating room staff roles.

Pre-intervention evaluation period

Following Session 1, Data Collection, a formative simulation was run by creating a situation necessitating the performance of an emergency undocking. The da Vinci Si system was docked to a modified ZOE Gynecologic Simulator by Gaumard® and ESPs were situated in the operating room as during routine gynecologic robotic surgery. The provider was read the clinical scenario while seated at the da Vinci surgeon console and oriented to the room. Upon taking control of the instruments, a vessel within the torso was designed to begin bleeding, resulting in massive hemoperitoneum. The ESPs in place were instructed to follow commands based on the discretion of the surgeon. The simulation terminated following access to the patient and initiating laparotomy.

Educational intervention

**Figure 3:**
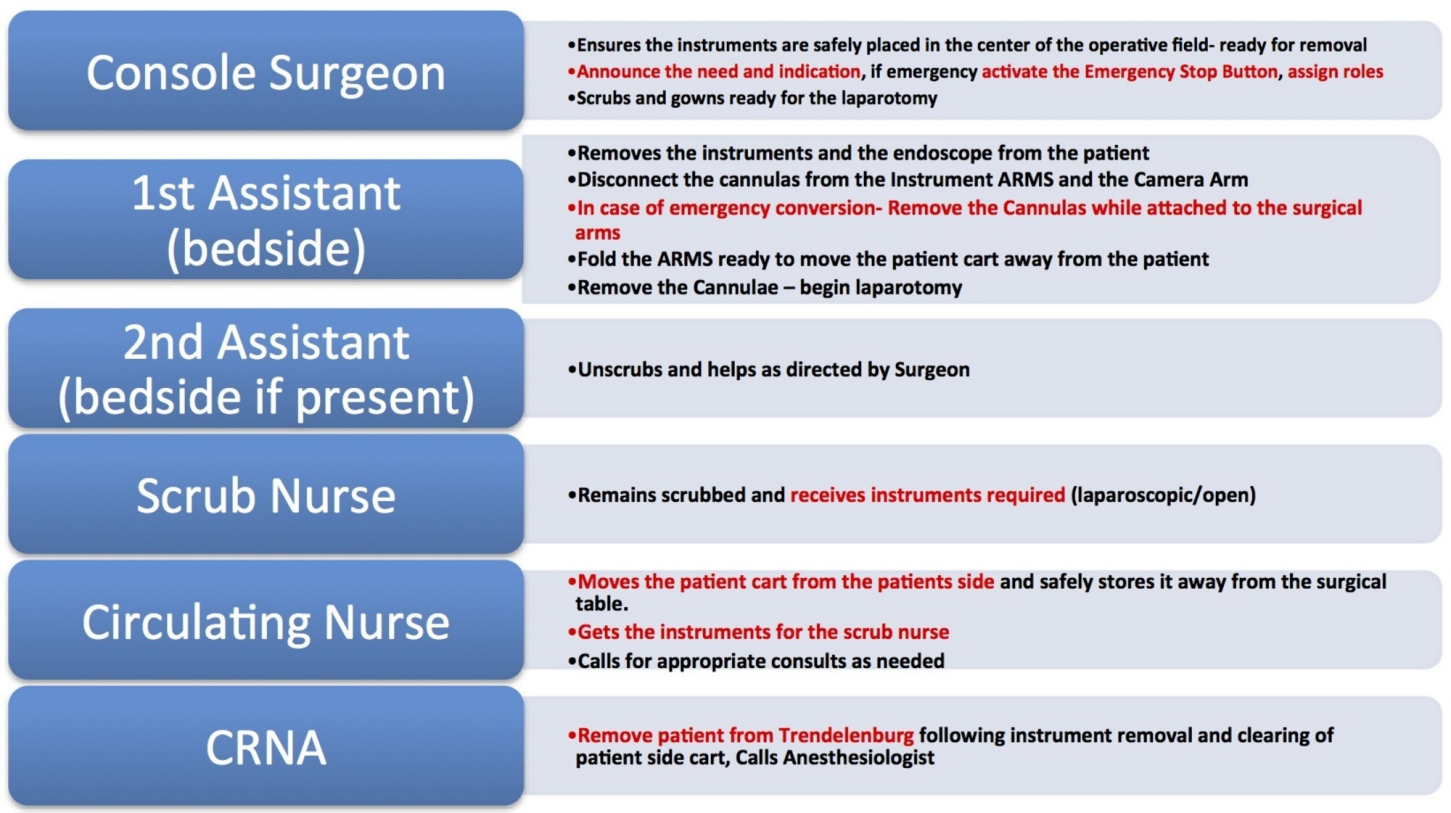
Emergency undocking team roles

Following the formative simulation, a debriefing individualized to the learner's performance (based on critical actions) was performed. This included a short presentation, and demonstrating the proper technique for emergency undocking with an emphasis on operating room personnel roles (Figure 3).

After completing the initial formative simulation, the learner was presented with didactic materials on emergency undocking to review independently over the next two weeks. After two weeks from the initial simulation, a review session took place to answer any questions regarding the didactic material, and learners were offered the opportunity to further practice the acquired skills. All learners participated in the review sessions, but no learners scheduled additional practice times.

Post-intervention evaluation

Four weeks from the initial simulation, a summative simulation was performed. The time from the identification of a major vessel injury until performing laparotomy was timed again, and successful completion of critical actions recorded and graded by a clinical expert. The surveys conducted prior to Session 1 were again collected for evaluation.

Data methods

Data were entered into an Excel database, which was imported into SPSS v24.0 software (IBM Corporation, Armonk, New York, US). Descriptive summaries were then calculated for the study cohort (n=21) at the baseline (prior to simulation) and post-simulation time points. Descriptive summary measures consisted of the mean and median as measures of centrality and the standard deviation, interquartile range (IQR), and range as measures of dispersion. Changes in the study outcomes (calculated as post-simulation value minus baseline value) were also summarized for the study cohort. Statistical tests for median change equivalence to zero were performed using the Wilcoxon signed rank test with p<0.05 considered statistically significant via two-sided testing.

## Results

**Figure 4:**
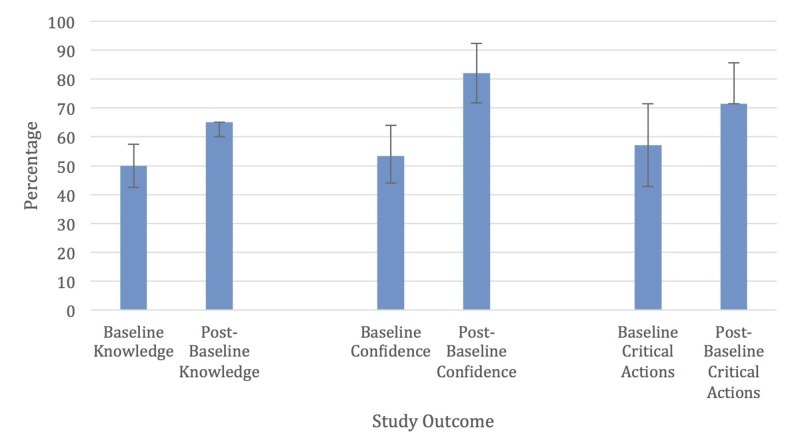
Study outcomes Study outcomes expressed as a percentage of the maximum score: median with error bars extending to the 1st and 3rd quartiles.

Column graphs of the median with the first and third quartile values for the baseline and post-simulation study outcome values were performed using Excel (Figure 4).

For these graphs, the study outcome values were expressed as a percentage of the maximum value, e.g., baseline median knowledge score of 10 correct out of 20 total questions was expressed as 50%.

When comparing the performance of all participants from their formative and summative simulations, the median increased from four to five out of a total of seven critical actions. The participants significantly increased the number of critical actions completed during their summative simulation by a median of one (p=0.002).

The overall average time to complete the emergency undock was calculated separately for the formative and the summative simulation. The mean pre-intervention time in seconds was 139 and post-intervention was 89. This was also found to be statistically significant (p<0.001).

Before training, the total baseline confidence had a median of 40 out of 75. Post-curricular baseline confidence had a median of 61.5 out of 75. This resulted in a median change of confidence of 18 (p=0.003).

Participants had a baseline median knowledge score of 10 correct responses. The post-curricular median knowledge score was 13. The overall change in knowledge was three questions (p=0.001).

## Discussion

Following participation in this curriculum, the surgeons demonstrated an increase in confidence, knowledge, and critical actions [14]. There was a significant improvement in the baseline level of knowledge (p=0.001). The results also showed improvements in the confidence of surgeons in the setting of an emergency undocking (p-value=0.003). The results of our study suggest that allowing robotic surgeons to engage in hands-on practice with the use of simulation during their training may improve familiarity with emergency protocols and improve confidence [14]. Mean undocking times were improved by -56.5 seconds (p-value<0.001) and there was an improvement in critical actions performed (p-value=0.002). It appears from the results that simulation training improves a robotic surgeon’s ability to both recognize the need for an emergency undocking and lead an interprofessional team during an emergency undocking [14]. This improvement could have life-saving implications.

Further, our results suggest that the level of experience of the robotic surgeon does not appear to have an effect on the overall gain in knowledge, change in confidence, or increase in critical actions performed. These comparisons should be cautiously interpreted due to the potential type II error caused by the small sample size and non-parametric testing procedure. These results, however, correlate to a study by Meier et al. investigating the learning curve of incorporating robotic surgery simulator training with surgeons of different ages and prior expertise, which demonstrated that, regardless of the level of training, all learners can benefit from training via robotic simulation [15]. Of note, the majority of attending physicians reported during the debriefing that had the bedside assistant been a resident physician, the surgeon would have instructed the resident to begin laparotomy earlier. This may have led to a shorter time to employ life-saving interventions.

There were a number of limitations to our study. The participants underwent training in the curriculum at a single institution and, although the participants represented a wide variety of subspecialties and training levels, all were within the field of gynecology. Additionally, we had a limited number of participants, which could affect the generalizability of the data. This also constrained our ability to further analyze the data beyond what was reported. In addition, learners were aware that the curriculum emphasized the need to undock the robot in a timely fashion, which may have influenced the improvement noted in our results. Finally, there was only a single fellowship-trained robotic surgeon grading critical actions, who was not blinded to formative versus summative encounters.

## Conclusions

This curriculum resulted in increased confidence and knowledge and demonstrated enhanced competence in robotic surgeons in performing an emergency undocking. Utilizing this curriculum can allow for the standardized training of robotic surgeon operators. Future studies are needed to evaluate efficacy in other fields beyond minimally invasive gynecology.
